# Knowledge, attitudes and perceptions of AI in medicine among Indian doctors

**DOI:** 10.6026/973206300211061

**Published:** 2025-05-31

**Authors:** Chidurala Rahul, Shriraam Karunakaran, Venkatesh Karthikeyan, Suresh Varadharajan, Swetha Chandru

**Affiliations:** 1 Department of Community Medicine, Sri Ramachandra Medical and Research Hospital, Porur, Chennai-600116, Tamil Nadu, India; 2 Department of Biochemistry, MVJ Medical College and Research Hospital, Bangalore-562114, Karnataka, India; 3 Department of Community Medicine, AIIMS, Patna-801507, Bihar, India

**Keywords:** Artificial Intelligence (AI), medical students, healthcare, knowledge attitudes perception

## Abstract

Artificial Intelligence (AI) is revolutionizing healthcare. However, there is a significant lack of understanding among medical practitioners on AI. Therefore, it is of interest to evaluate the knowledge, awareness, experience and perceptions regarding AI among 250 medical students, doctors and members of the Indian Medical Association (IMA). The questionnaire based survey revealed that though the participants were well aware of the general AI concepts, not even half felt that it would be helpful for diagnosis and treatment purposes. Also, 82.9% (n=207) reported not taking any formal courses on AI, which again emphasizes a learning gap. Results suggest better training of AI within the medical education framework to prepare healthcare professionals to face imminent trends.

## Background:

Innovations in artificial intelligence are giving an entirely new complexion to future healthcare scenarios and almost every aspect of the medical field is going to be transformed. AI is primarily the driving force behind advancements in fields like medical images, performing robotic surgery and developing personal treatment methods [1, 2]. AI can be software or hardware based. Electronic Health Records (EHRs) are analyzed using neural networks in software AI, while hardware AI includes robot-assisted surgery and procedures [3]. AI is used in drug discovery, disease diagnosis, healthcare planning, monitoring, digital consulting, surgery, data management, personalized treatment and decision-making [4]. The advancement of AI can change healthcare much and even disrupt medicine. A plethora of literature has been written about artificial intelligence in the medical field [5, 6, 7-8]. To date, only a few studies have been conducted regarding the awareness of artificial intelligence in medicine among physicians and medical students [9]. Therefore, it is of interest to evaluate the knowledge of artificial intelligence (AI) in medicine among medical students and doctors and their perceptions regarding its use in medicine.

## Materials and Methods:

This study was conducted at Sri Ramachandra Medical College and Research Institute from 10/08/23 to 10/08/24, with a sample size of 250. The questionnaire consisted of 24 questions that addressed key concepts in AI in the medical field. The inclusion criteria were healthcare professionals and students. This study excluded non-healthcare professionals. After obtaining ethics committee approval, the questionnaire was shared with the participants through Google Forms.

## Study procedure:

This was a cross-sectional questionnaire-based study. Regarding questionnaire validity and reliability, a structured questionnaire was developed following a thorough literature review conducted initially by the chief investigator and research papers were selected for further discussion among the research team. All opinions, thoughts and concerns about the proposed study were considered throughout the planning stage. An initial questionnaire draft was created after the research team thoroughly reviewed all the selected papers. The list of healthcare professionals was obtained from the IMA register and participants were selected by stratified random sampling. The participants would be contacted over the phone and after obtaining verbal consent, a link to an online Google form would be sent through email or to their phone number. The form would initially contain the participant information sheet followed by an informed consent after the participant accepts the informed consent online; the web page would lead to a structured questionnaire administered through the same Google forms. A group of medical professionals reviewed individual survey items and agreed on their clarity and importance. The physicians participated voluntarily. The questionnaire was drafted in English. The questionnaire comprised 18 statements, sectioned into five events: demography, knowledge, awareness, perception and opinion.

## Statistical analysis:

The responses from the online form would be exported to Excel and then analyzed using Epi Info 7.2 for Windows (https://www.cdc.gov/epiinfo/pc.html). The descriptive analysis of participants would be followed by calculating the proportion of participants answering each question appropriately and the perceptions of using AI in healthcare. A sub-group analysis would be done based on qualification and/or experience. Statistical analysis involved an anonymous questionnaire distributed via Google Forms to all participants.

## Results:

A total of 250 participants filled out the questionnaire. The mean age of participants was 23.2 years (SD = 4.2). More females (60.4%, n= 151) were in the study (Figure 1 see PDF). As the forms were online, participants from various parts of the country responded to the questionnaire. Most participants were from the Southern region (64.7%, n=162), followed by Western (18.5%, n=46), Northern (9.5%, n=24), Central (3.2%, n=8), Eastern (2%, n=5) and North-East (2%, n=5). The undergraduate medical students comprised 49% (n=170) of the participants (Figure 1 see PDF). The majority (82.9%, n=207) of the participants did not attend any course on artificial intelligence.

## Benefits of AI in Healthcare (as noted by participants):

Participants frequently highlighted several key advantages of AI in healthcare, including its ability to improve diagnostic accuracy and speed up diagnosis and treatment, which they believe will streamline patient care. Many pointed out how AI increases the processing efficiency of complicated data, hence relieving pressure on healthcare professionals. Participants commented on how AI reduces human errors to ensure reliable results. Participants further mention that AI increases time and cost efficiency, particularly in administrative work, drug discovery and research. Finally, many participants spoke on how AI significantly supports healthcare professionals while improving the decision-making process and, consequently, the management of patients.

## Knowledge of concepts related to Artificial intelligence:

From the knowledge domain of the questionnaire, we inferred that the maximum number of respondents had good knowledge of AI concepts (Figure 2 see PDF). Most knew datasets, algorithms, machine learning and deep learning. Some had ideas of Personal Data Protection (53.2%, n=133), Digital Information Security in Healthcare Act (47.2%, n=118), human-in-the-loop concepts (34.8%, n=87), DISHA Bill (34%, n=85), SPIRIT-AI and CONSORT-AI (20%, n=50) as seen in Figure 2 - (see PDF).

## Knowledge of utilization of Research involving AI:

One forty-three (57.2%) of the participants felt that a person with expertise in AI is required to research human participants using AI. The majority (94.2%, n=236) of participants responded that consent is required to collect data on name, address and clinical records. Around 85% (n=213) of participants appropriately responded that a participant could withdraw from the study at any time. Only 6.9% (n=17) responded that the validity of an algorithm should be assessed on a different dataset than the one used to create it. Only 6% (n=15) knew that data should be collected from people with different socio-demographic profiles to develop the AI algorithm. None of the participants responded to questions related to the effect of skewed training datasets and data anonymization. Around 40% (n=100) of the participants knew about the Digital Information Security in Healthcare Act.

## Awareness:

More than half of the participants were aware of certain key concepts related to AI. The majority (80.3%, n=201) knew that algorithms are an essential component of AI and that datasets are required to develop AI (72%, n=180) (Figure 3 see PDF). The proportion of correct responses was higher for consultants/faculty than for other groups.

## Perception:

In this study, the responses of 250 participants were measured regarding the perceptions of the use of AI in medical practice (Figure 4 see PDF) in a few significant areas and found that, while 51% (n=128) were positive toward application at diagnosis and treatment, 36% (n=90) were neutral and 13% (n=32) opposed using AI. When asked about AI's potential to reduce medical errors and improve patient outcomes, 60% (n=150) agreed, 30% (n=75) were uncertain and 10% (n=25) disagreed. Similarly, 64% (n=160) of participants believed that AI-powered tools could significantly enhance diagnostic accuracy, with 29% (n=72) expressing uncertainty and 7% (n=18) disagreeing. The notion of AI replacing doctors in the future was primarily rejected, with 61% (n=153) either strongly disagreeing or disagreeing, while only 16% (n=40) agreed and 23% (n=57) remained neutral. Regarding the inclusion of AI in the medical curriculum, 44% (n=110) supported it, 34% (n=85) were neutral and 37% (n=92) were opposed. Finally, 47% (n=117) of respondents felt that AI is essential for the future of medicine, with 23% (n=58) holding a neutral stance and 29% (n=72) disagreeing. Overall, the findings suggest cautious optimism toward AI's role in medicine, with notable skepticism surrounding its potential to replace doctors and its place in medical education. The findings are summarized in Figure 4 - (see PDF).

Participants believe that the practice of ethics with AI in healthcare should include knowledgeable consent and complete confidentiality of patients. Country-specific legislation and codified rules and regulations regarding the use of AI, regular monitoring and good cyber security should be implemented so that errors are ruled out and there is no breach of confidence. Human oversight was principal to complement AI instead of becoming dependent on technology. These recommendations advised that AI ethics be included in curricula for medical education, decisions made by AI should be again verified through multidisciplinary teams, high levels of transparency should be maintained about the processes undertaken by AI and applications should be well defined.

[1] A person with expertise in AI is required for research on human participants using AI;

[2] Consent is not required for taking records like name, age, address and clinical information from case sheets;

[3] Once the participant has enrolled in the study he/she cannot withdraw from the study at a later point in time;

[4] Additional consent may be obtained from participants if data will be used at a later point in time;

[5] The validity of an algorithm should be assessed on a different dataset than the one which was used to create it;

[6] To get the data for developing the algorithm people of different socio-demographic profiles need to be selected to develop a good algorithm;

[7] Skewed training datasets can result in data bias;

[8] A technique to protect confidentiality while before data sharing is through data anonymization;

[9] The errors occurring due to hardware or AI software during treatment will be taken responsibly by the AI developer;

[10] Access to AI technology for the underprivileged should be restricted to prevent occurrence of risk;

[11] The advantage in AI research involving human participants is that no specific permission is required from Government to share data with International collaborators.

## Experience:

The participants had varying usage frequencies of AI-based tools in their practice of medicine or research (Figure 5 see PDF). The majority, 45% (n=123), admitted using AI tools rarely. Among the respondents, 25% (n=63) reported using AI tools regularly, while smaller portions occasionally (15%, n=37), frequently (12%, n=30) and very often (3%, n=7). Regarding the frequency of use of AI tools, 35% (n=87) said they had never used such tools, 23% (n=58) said they used them monthly, 17% (n=43) admitted to using them weekly, 17% (n=42) two to three times a week and only 8% (n=20) use them daily. However, a majority of 92% (n=230) expressed interest in updates on this study on AI, while 8% (n=20) did not express a particular interest. Evidence indicates that even though AI diffusion is limited, there is already considerable interest in knowing more and more about the science of progress in the discipline. Most said they had minimal or no experience with AI-based healthcare technology, especially VR or AR, but some found it beneficial for learning. Those who had used such tools, especially for learning anatomy, reported that those tools were exciting and valuable in offering a view of complex structures and memorability and attracting the learner. Tools like AR Anatomy apps and Simulation labs were lauded for enhancing understanding, providing ethical alternatives to traditional methods and improving precision in medical procedures. Meanwhile, others used AI tools, including ChatGPT, to retrieve quick, accurate information while studying medicine, though some noted limitations in learning concepts visually and accessing research articles. Altogether, the use of AI in medical education and practice seems promising and worthwhile in the future.

## Discussion:

Knowledge, awareness, perception and experiences of artificial intelligence in medicine among medical students, doctors and faculty members were assessed in this study. The results were evaluated to understand whether the participants had a basic understanding of the concepts surrounding algorithms, machine learning, *etc.*; however, most participants had never received formal training in AI. Over 82.9% (n=207) of the participants revealed that they had never attended a course about AI. In addition, though 51% (n=127) responded positively about the capability of AI in diagnosis and treatment, an appreciable proportion of respondents remained neutral or skeptical, mainly regarding AI in replacing doctors. Such results give a feeling that though there is an increase in awareness of AI in health care, uncertainty prevails in various sectors on practical applications due to less exposure and training.

The study validates previous studies in that healthcare professionals know AI's potential but often lack the training to use it fully. For example, a study concluded that although most doctors believe AI can improve the accuracy of diagnoses, they are not prepared to adopt AI into their practices [10]. Research involving medical students in Jordan demonstrated moderate AI knowledge, with students recognizing AI's value but expressing skepticism about its ability to replace human educators [11]. Cross-sectional studies in Pakistan found that while healthcare professionals and students recognized AI's potential in improving healthcare efficiency and patient outcomes, only a small fraction were aware of its medical applications. The lack of formal AI education in medical curricula was a common barrier to its widespread adoption [12, 13]. Another study concluded that formal education for AI is a significant barrier to its adoption in clinical practices [14]. Our results also highlight this gap, which means doctors know what AI can do but lack the education to bring AI into medical practice. People feel both hopeful and uncertain about AI in healthcare. Existing literature indicates that AI has the potential to improve diagnostic accuracy and reduce human errors in medical imaging [15]. They also believe AI has the potential to transform healthcare by enhancing decision-making and simplifying complex processes [16]. There is skepticism about AI replacing physicians; only 16% (n=55) think AI might fully replace doctors. This is in accordance with a study that recognized that although AI is known to assist in the better delivery of services by healthcare providers, there is an outright refusal to consider the complete takeover of human functions by AI [17]. The use of AI-based tools in practice differed between the medics and researchers. A study conducted among doctors in Bahraich, India, found that while many had a moderate to high understanding of AI, only a small number had integrated it into their medical practice [18]. Another study at Sultan Qaboos University found that while medical students see AI as a promising tool in healthcare, many feel unprepared to use it due to a lack of formal education on the subject [19]. However, an interest in AI continues to be shown as most participants are interested in recent updates. Using AI tools such as AR Anatomy apps and Simulation labs improved the understanding of anatomy. It brought up alternative ethical means of gaining knowledge over the traditional methods that were commonly used. Hence, these findings fall in line with the studies that indicate. At the same time, the adoption of AI is relatively low in health care; there is excitement in the ability to enhance learning, precision in medical procedures and overall practice [20, 21]. These implications are significant in medical education. With AI in health care, there is a need to prepare future medical professionals for AI-driven environments. Of course, this would require incorporating AI training into the medical education curriculum to facilitate the gap between awareness and practical implementation of AI. Another study concluded that early exposure to AI is essential so that medical trainees become confident in using AI technology in the clinical setting [22]. Our study confirms this finding, with 92% (n=230) of our respondents stating an interest in receiving more information about AI. Many biases plague questionnaire-based studies, such as social bias, where the respondents give answers that they perceive to be socially desirable rather than their actual beliefs; self-reporting biases due to respondent fatigue and lack of understanding of the context; and reliance on memory and perception of participants that introduce recall and subjective interpretation biases. In conclusion, the study emphasizes the critical need for AI education in medical schools. As artificial intelligence continues to revolutionize healthcare, it is crucial to prepare future healthcare professionals with the knowledge and skills to use AI effectively. Bridging the gap between awareness and practical application will be critical in ensuring that AI fulfills its potential to improve patient care and medical research.

## Conclusion:

The data indicates that medical professionals have knowledge, awareness, perception and opinion of Artificial Intelligence (AI) in Medicine, which merits further insight. We show that most medical professionals were not regularly exposed to AI tools. If this issue is resolved, AI will significantly influence healthcare more.
